# Prey removal in cotton crops next to woodland reveals periodic diurnal and nocturnal invertebrate predation gradients from the crop edge by birds and bats

**DOI:** 10.1038/s41598-021-84633-8

**Published:** 2021-03-04

**Authors:** Heidi L. Kolkert, Rhiannon Smith, Romina Rader, Nick Reid

**Affiliations:** Ecosystem Management, School of Environmental and Rural Science, University of New England, Armidale, NSW 2351 Australia

**Keywords:** Agroecology, Ecosystem services

## Abstract

Factors influencing the efficacy of insectivorous vertebrates in providing natural pest control services inside crops at increasing distances from the crop edge are poorly understood. We investigated the identity of vertebrate predators (birds and bats) and removal of sentinel prey (mealworms and beetles) from experimental feeding trays in cotton crops using prey removal trials, camera traps and observations. More prey was removed during the day than at night, but prey removal was variable at the crop edge and dependent on the month (reflecting crop growth and cover) and time of day. Overall, the predation of mealworms and beetles was 1-times and 13-times greater during the day than night, respectively, with predation on mealworms 3–5 times greater during the day than night at the crop edge compared to 95 m inside the crop. Camera traps identified many insectivorous birds and bats over crops near the feeding trays, but there was no evidence of bats or small passerines removing experimental prey. A predation gradient from the crop edge was evident, but only in some months. This corresponded to the foraging preferences of open-space generalist predators (magpies) in low crop cover versus the shrubby habitat preferred by small passerines, likely facilitating foraging away from the crop edge later in the season. Our results are in line with Optimal Foraging Theory and suggest that predators trade-off foraging behaviour with predation risk at different distances from the crop edge and levels of crop cover. Understanding the optimal farm configuration to support insectivorous bird and bat populations can assist farmers to make informed decisions regarding in-crop natural pest control and maximise the predation services provided by farm biodiversity.

## Introduction

Finding solutions to reduce pesticide use in crops and enhance the abundance of natural enemies of crop pests in agroecosystems is a pressing global issue. Worldwide, pests and diseases are responsible for an estimated 20–40% of crop yield losses^1^. Natural enemies can suppress insect pests below economic thresholds and therefore provide a viable alternative to pesticide use^2,3^. Natural enemies of pests, including vertebrate and invertebrate insectivores (birds, bats and arthropods) and insect parasitoids, provide a service worth billions of dollars to global agriculture^4–6^. As such, natural pest control is one of the most important and economically significant ecosystem services to agriculture^7^. Yet, many aspects of natural pest control in crops remain unclear. In particular, the identity of the vertebrate predators providing this service is generally unknown and the efficacy of predation inside crops at increasing distances from the crop edge is poorly understood. This information is important for farmers who implement habitat modifications under agri-environmental schemes for natural pest control to determine how far inside crops the benefits of non-crop habitat may extend.

Insectivorous birds and bats protect crops from pest arthropod damage and are economically important natural enemies in agricultural systems^8–14^. Maintaining non-crop habitat for these natural enemies of crop pests can therefore provide both ecological and economic benefits^15^. Birds and bats have high energetic demands due to flight, and consume large amounts of prey to fuel high rates of energy expenditure^16,17^. Globally, 400–500 million tonnes of arthropods are consumed by birds annually, with 50% of birds considered insectivorous and an additional 25% of birds consuming invertebrates occasionally^18^. The efficacy of insectivorous birds in suppressing arthropods in agricultural and forest systems is well documented^19^. Bird-mediated reductions in arthropod abundance in crops typically vary from 20 to 70%^12,19–21^. For example, insectivorous birds significantly reduced densities of large arthropods and damage due to foliage-chewing insects by 46% in cacao crops^21^, and reduced infestation of the coffee berry corer (*Hypothenemus hampei*) by 50%^12^. Similarly, predation of the corn earworm by *Tadarida brasiliensis* (Brazilian free-tailed bat) is estimated to increase corn crop yields by 1.4%, with bats tracking and exploiting moth irruptions^22,23^. In inland eastern Australia, agricultural pests comprised 65% of the identified diet of insectivorous bats in cotton crops and were the six most frequently consumed prey items, suggesting a significant pest regulation service^24^.

Much of the work on insect pest control by insectivorous birds and bats has occurred in tropical forest and agricultural production systems^13,25–27^, with few studies in subtropical or temperate agroecosystems, aside from cotton crops in Northern America^28^, macadamia orchards in South Africa and Australia^29^, wheat^11,30^ and brassicas^31,32^. Studies attempting to separate and quantify the insect pest control service provided by birds and bats use exclosures^12,13,25,33^, or plasticine model caterpillars as a proxy for lepidopteran control^34^. Although these studies generally show that birds suppress more arthropods than bats, few link this service to pest consumption rates and some, but not all, infer insect predation via reduced leaf herbivory and increased yields^34–37^. Furthermore, the contribution of small nocturnal mammals to insect pest control is often overlooked, yet a growing body of evidence suggests that they provide an important pest regulation service^38,39^.

Diurnal and nocturnal vertebrate predators suppress different groups of arthropods and thus impact trophic processes (such as leaf herbivory) differently^12^. Insectivorous bats mostly target soft-bodied Lepidoptera above crops or glean from leaf surfaces^22,23^, whilst small insectivorous passerines forage in the shrub layer from the ground up and consume mainly ants, wasps, flies and beetles with a mean prey size of around 4–8 mm^40–43^. Larger passerines consume a range of small and large invertebrates and provide effective pest control of fossorial scarab larvae^44^ and above-ground pests including crickets, grasshoppers and beetles. Yet, remarkably little information exists about the identity, predation rate or potential pest control service provided by the range of vertebrate fauna present in any agricultural system.

Crop edge habitats provide a different physical and microclimatic environment compared with crop interiors. The activity of vertebrate predators is often greater at crop edges than in crop interiors^45,46^, with a concomitant increase in predation of invertebrates close to the crop edge^47,48^. Crop edges provide flyways for foraging birds; less complex echolocation for bats to navigate, target and catch prey than adjacent non-crop woody vegetation; access to abundant crop insect prey resources, and reduced predation risk compared to crop interiors. Crop edges are thus optimal foraging habitat for insectivorous birds and bats in agricultural mosaic landscapes due to access to prey^45,49^. Under optimal foraging theory (OFT), foraging decisions made by an animal to maximise energy consumption in the least amount of time possible are constrained by the detection, handling and capture of their prey and the risk of predation of the foraging animal itself^50^. Thus, foraging over open crops at increasing distance from the crop edge—woody vegetation interface is a trade-off between flight capabilities, foraging behaviour and the risk of predation. In terms of bats, echolocation constraints imposed by habitat clutter determine whether they usually locate and capture prey in narrow or cluttered flyways, at habitat edges, or in open habitats^51,52^. Whilst edge-space bats typically forage along vegetation interfaces in open areas such as inside crops edges^53,54^, most bats show some plasticity in foraging strategy and echolocation behaviour^54,55^, suggesting the potential adaptive capability for bats to forage away from the crop edge in the crop interior.

Cotton (*Gossypium* spp.) is a fibre crop and the most valuable non-food agricultural commodity worldwide^56^. However, cotton is also the second-top global host for arthropod pests, with several cotton pests considered major pests worldwide^57^. Hence, the control of insect pests (particularly *Helicoverpa* spp., cotton bollworms) is a major issue. Integrated pest management (IPM) in Australian cotton crops typically includes encouraging a mix of beneficial arthropods (such as predatory beetles and parasitic wasps)^6,58–61^, transgenic crop biotechnology and minimising pesticide use. The effectiveness of IPM in Australian cotton crops would be enhanced with information about the diversity of vertebrate predators providing natural pest control. Cotton-growing regions in northern New South Wales (NSW) and southern Queensland host an abundance of naturally occurring vertebrate predators more than one-third of all Australian land birds^62,63^, over one-quarter of insectivorous bat species (H. Kolkert et al., unpublished data) and several threatened insectivorous marsupial dasyurids and other small native rodents—that likely play an integral role in suppressing insects in crops, but are underutilised in IPM programs. In this study, we identified vertebrate predators and assessed predation rates in cotton crops across the growing season. We used a combination of prey removal trials and camera traps in cotton crops at incremental distances from the crop edge as well as bird census, to investigate predation rates of diurnal and nocturnal vertebrate predators on two types of sentinel prey (live mealworms and dead beetles). Ants interacted with prey at feeding stations and were identified as possible important predators and were also included in our analysis. We asked four questions: (1) Which vertebrate taxa are present and remove insect prey in cotton crops? (2) Is predation greater by day or by night? (3) Does proximity to the crop edge influence prey removal? (4) How do prey removal rates change over the cotton growing season?

## Results

### Mealworm removal

**Figure 1:**
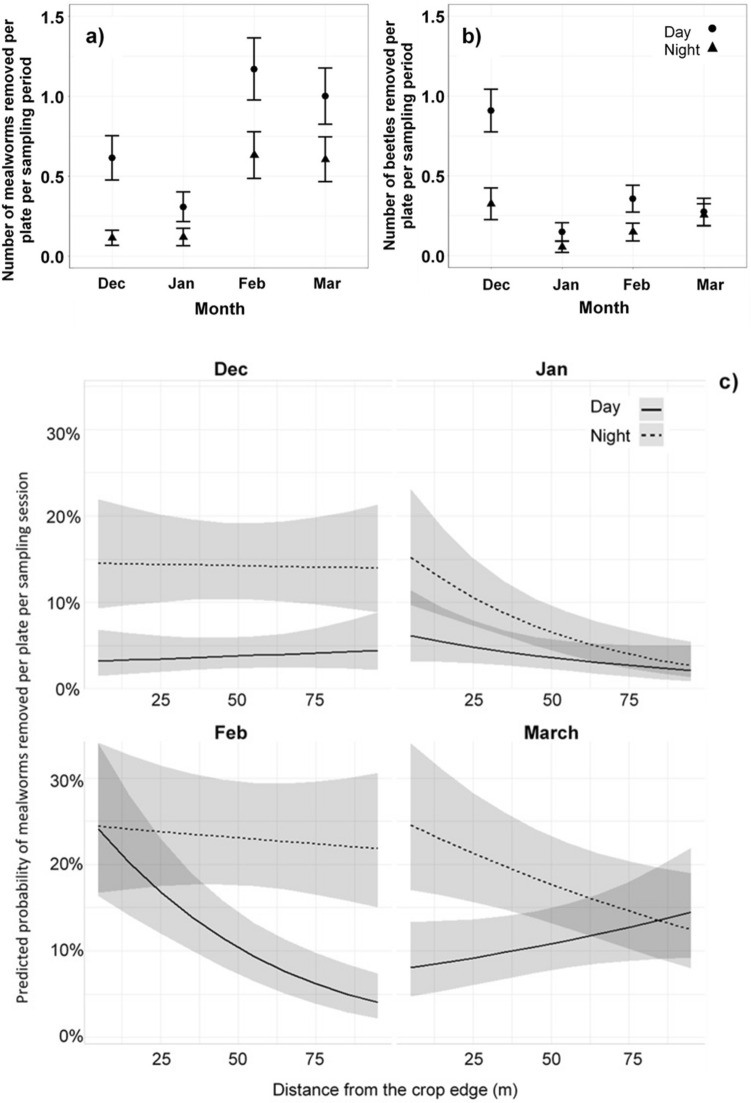
Mean number of (**a**) mealworms and (**b**) beetles removed, averaged over feeding station and sampling session by month (with 95% confidence intervals) and (**c**) predicted removal of mealworms at increasing distances from the crop edge by time of day (day vs night) and month, showing the 3-way interaction. Shaded areas represent 95% confidence intervals.

Around 11% (1814) of mealworms were removed out of the 16,300 provided, ased on 3260 sampling observations across an Australian summer cotton growing season. Mealworms were 12.5% more likely to be taken by day than night (*Odds Ratio* [OR] 0.88, Supplementary Table S2 online). More mealworms were also removed later in the season (Fig. 1a). In February and March, diurnal mealworm predation doubled, whilst nocturnal mealworm predation increased five-fold compared to December and January.

Distance from the crop edge significantly affected mealworm predation but depended on month (Fig. 1a) and time of day (Table 1, Fig. 1c). Over the whole season, 3–5 times more mealworms were removed at the crop edge than at 95 m inside the crop during the night and day, respectively (Fig. 1c and Supplementary Table S3 online). Predation on mealworms did not vary with distance into the crop in December (Fig. 1c and Supplementary Table S3). In January, predation on mealworms significantly declined with increasing distance from the crop edge and similarly by night in February (Fig. 1c and Supplementary Table S1). However, at night in March, this trend reversed and more mealworms were removed in the crop interior than edge (Fig. 1c and Supplementary Table S3).

**Table 1:** Analysis of Deviance Table (Type III Wald chi-squared tests) for mealworm and beetle predation, showing the chi-squared statistic (Chisq), degrees of freedom (Df) and p-value.

Factors	Mealworm removal			Beetle removal		
	Chisq	Df	p-value	Chisq	Df	p-value
(Intercept)	40.174	1	**0.000**	0.459	1	0.498
Time of day	12.926	1	**0.000**	24.054	1	**0.000**
Distance	0.019	1	0.890	1.746	1	0.186
Month	7.707	3	0.052	48.702	3	**0.000**
Time of day: Distance	0.355	1	0.551	0.202	1	0.653
Time of day: Month	14.734	3	**0.002**	15.210	3	**0.002**
Distance: Month	10.898	3	**0.012**	2.316	3	0.509
Time of day: Distance: Month	21.463	3	**0.000**	1.785	3	0.618

Significant probabilities are shown in bold.

### Beetle removal

Around 8% (1254) of beetles were removed of the 15,060 provided. Beetles were 13 times more likely to be removed during the day than at night, holding all other variables constant (*OR* 14.00, Supplementary Table S2 online). However, the interaction between time of day and month had a significant effect on beetle predation (Table 1). Predation on beetles was greatest by day in December, with 5-times the number of beetles removed than at night in December or in other months either by day or night (Fig. 1b). Significantly more beetles (around 2.5-times more) were removed by day than night in January and February, but there was no difference in March (Fig. 1b and Supplementary Table S1). The effect of distance to the crop edge on beetle predation was not significant (Table 1).

Compared to beetles, mealworms were 87% more at risk of predation *(OR* 0.13), holding all other variables constant (Supplementary Table S2 online). There was a high probability that a predator consumed zero beetles in the experiment (predicted probability 26%, zero inflation model).

### Likely predators

#### Camera images

In total, 972 camera images with vertebrate species present were tagged (Table 2) from a total of 6449 day-camera hours and 5209 night-camera hours. Camera images revealed that prey was removed from the feeding stations by a variety of fauna (Table 3a and Supplementary Fig. S1 online). Five bird species common in agricultural landscapes were recorded, including two nocturnal and three diurnal species: eastern barn owl (*Tyto alba*), southern boobook (*Ninox boobook*), willie wagtail *(Rhipidura leucophrys*), Australian magpie (*Gymnorhina tibicen*) and pied butcherbird (*Cracticus nigrogularis*). Nocturnal mammals could not be confidently identified to species level and were grouped as either insectivorous bats or other small mammals (Table 3a).

**Table 2:** The number of tagged images from camera traps, the visitation rate expressed as camera trap images per hour (CT/h) and the visitation length expressed as an activity index (AI/h).

	Number of tagged images		Visitation rate		Visitation length	
	Daylight	Nocturnal	Daylight CT/h	Nocturnal CT/h	Daylight AI/h (min s)	Nocturnal AI/h (min s)
**Month**						
December	61	393	0.93	6.34	4.31	0.13
January	2	430	0.03	23.76	0.00	0.24
February	12	48	0.15	0.99	0.42	0.01
March	14	12	0.15	0.14	0.04	4.61
**Total**	**89**	**883**	*0.32	*7.81	*1.19	*1.25
**Distance from crop edge by month**						
**Close**	**55**	**712**	*0.22	*6.88	*0.95	*0.07
December	39	316	0.69	5.14	3.80	0.05
January	2	390	0.03	22.31	0.00	0.22
February	6	6	0.04	0.06	0.00	0.00
March	8	0	0.11	0.00	0.00	0.00
**Far**	**34**	**171**	*0.10	*0.93	*0.24	*1.18
December	22	77	0.25	1.20	0.51	0.08
January	0	40	0.00	1.45	0.00	0.01
February	9	44	0.12	0.93	0.42	0.01
March	3	10	0.04	0.14	0.04	4.61
**Total**	**89**	**883**	*0.16	*3.90	*0.60	*0.62

Average visitation rate and length denoted with an asterisk.

Bold values indicate a significant p-value <0.05.

**Table 3:** Fauna species recorded.

(a) Fauna	Day				Night				Total
Aves	Dec	Jan	Feb	Mar	Dec	Jan	Feb	Mar	
Australian magpie (*Gymnorhina tibicen*)	61	2	–	–	–	–	–	–	63
Southern boobook (*Ninox boobook*)	–	–	–	–	–	–	–	1	1
Eastern barn owl (*Tyto alba)*	–	–	–	–	–	–	1	–	1
Pied butcherbird (*Cracticus nigrogularis*)	–	–	–	6	–	–	–	–	6
Willie wagtail (*Rhipidura leucophrys*)	–	–	12	8	–	–	–	–	20
Mammals									
Insectivorous bat sp.	–	–	–	–	390	430	46	9	875
Other small mammals	–	–	–	–	3	–	1	2	6
Total	61	2	12	14	393	430	48	12	972

(b) Fig	Morning				Evening				Total
Fauna	Dec	Jan	Feb	Mar	Dec	Jan	Feb	Mar	
Golden-headed Cisticola (*Cisticola exilis*)	–	–	13	13	–	1	9	9	45
Inland thornbill (*Acanthiza apicalis*)	–	–	1	10	–	–	–	7	18
Australian magpie (*Gymnorhina tibicen*)	10	1	7	2	5	–	–	3	28
Nankeen kestrel (F*alco cenchroides*)	1	–	–	–	–	–	–	–	1
Pied butcherbird (*Cracticus nigrogularis*)	–	–	–	1	–	–	–	–	1
Red-browed finch (*Neochmia temporalis*)	–	–	–	9	–	–	–	–	9
Superb fairywren (*Malurus cyaneus*)	–	–	12	14	–	–	15	13	54
Willie wagtail (*Rhipidura leucophrys*)	–	1	7	–	1	1	3	1	14
Total	11	2	40	49	6	2	27	33	170

Number of (a) tagged images by time of day vs night and month (December to March) recorded by motion-activated cameras (b) fauna species recorded during 20-min observations of the sampling areas during morning and evening sampling sessions. Columns represent the abundance of species during the morning and evening sampling and during each month (December to March).

Australian magpies were the main predators during the day (and were the main consumer of beetles, H. Kolkert, pers. obs.) whilst camera traps (CT) revealed that insectivorous bats were the main predator in crops during the night (Table 3a). However, bats were not captured on camera gleaning experimental prey or landing on the feeding trays. Rather they were captured foraging above the cotton close to the feeding trays. Insectivorous bats were present in 10-times more images than diurnal fauna (birds), with most visitations (tagged images) occurring in December and January. At night, January had the highest visitation rate to feeding trays (~ 24 CT/h; Table 2).

In the day, December had the highest visitation rate and length of visits (measured as an activity index, 0.93 CT/h and 4 m 31 s AI/h; Table 2) with magpies responsible for nearly all the prey items removed from feeding stations, as indicated by camera trap images. Visitation length at night (in March) was strongly skewed by two incidents where a small scansorial mammal sat on the feeding station for several hours. During the day, twice as many visits were recorded close to the cotton edge than inside the crop (Table 2). At night, seven times more visitations were recorded close to the cotton edge than inside the crop. However, visitation length was the inverse, skewed by the lengthy small mammal visits. Hence, visitation length was not a reliable predictor of prey removal in this experiment.

#### Birds, scats and ant counts

**Figure 2:**
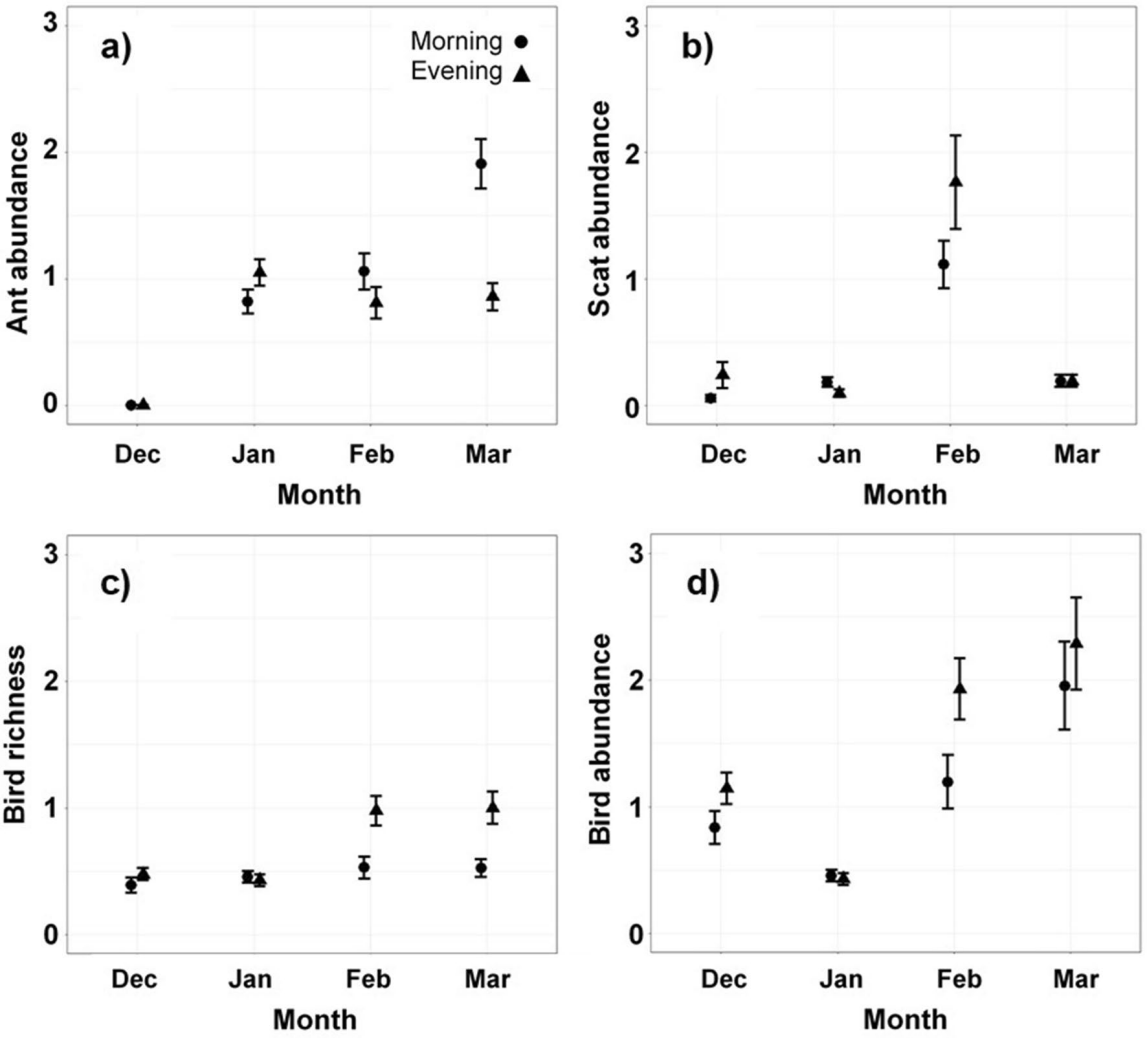
Mean (**a**) ant abundance, (**b**) scat abundance, (**c**) bird species richness and (d) bird abundance per morning and evening sampling session averaged by month with 95% confidence intervals.

Eight species of bird were recorded in the sampling areas, totalling 170 bird observations (Table 3b). Mean monthly bird richness, bird abundance, ant abundance and scat abundance during morning and evening censuses were greatest late in the season (February and March) compared to earlier in the season (Fig. 2). This pattern reflects the monthly pattern of mealworm predation (Fig. 1a). Potential predators and climate variables were significantly correlated with patterns of mean total prey removal, mealworm removal and beetle removal rates, but the relationships were weak (r ≤ 0.16, Table S4). Additional species of small insectivorous bird were noted inside cotton crops, but these were recorded outside the sampling areas (H. Kolkert, pers. obs.).

## Discussion

Understanding the optimal timing and location of beneficial insectivorous birds and bats can support decision-making to configure farm layout to maximise the insect pest predation services provided by farm biodiversity. In this study, we identified diurnal and nocturnal vertebrate insectivores that were present or removed experimental prey in a high-value commodity crop. A clear predation gradient from the crop edge was identified, but the gradient was only evident in some months. The variation in mealworm removal rates likely reflects predator foraging behaviours in response to crop cover versus open habitat, in-line with OFT^64^. For example, small insectivorous passerines (thornbills, cisticolas and fairywrens) are cover-dependent and were present in crops later in the growing season (Table 3b), when established cotton plants provided greater foliage cover, shelter, and protection from predators. Mature, shrubby cotton (mainly ~ 150 cm, but up to 200 cm high) facilitated small bird movement and access inside the crop, and best explained the lack of mealworm predation gradient from the edge into the crop during March. Increased foraging opportunities for cover-dependent species during February and March may also explain the higher mealworm removal rate (both by day and by night) than in other months. The lack of predation gradient in mealworm removal in December may be attributed to the absence of small birds traversing the sharp interface between crop edge habitat and the open field of young cotton. The low height and foliage cover of cotton plants (~ 30 cm high) in December also favoured larger birds of open habitat (Australian magpies) that are less sensitive to foraging away from the crop edge. These results support previous studies that identify shrubby habitat as a mediating factor for the movement of small birds across contrasting edge habitats in agricultural and forest systems^45,65^. It also highlights the importance of sufficient understorey cover in habitat immediately adjacent to the crop for edge-sensitive insectivorous birds and bats to facilitate movement and foraging in crops.

The removal of beetles primarily by the Australian magpie and peak beetle removal in December suggest that generalist predators suited to hunting in open habitat are less sensitive to crop edge proximity (moving throughout the crop), and explains the lack of an edge predation gradient early in the season. Increased foraging opportunities that benefit ground-feeding omnivores like Australian magpies occur early when ground cover is sparse. Therefore, our results suggest that predators favouring open foraging habitat benefit farmers during the initial stages of crop development (when bare soil is exposed). Small insectivorous birds did not consume beetles from feeding stations, to our knowledge. The size of the prey items offered may have excluded smaller predators from the experiment due to differences in prey size and thus availability to predators. This highlights the importance of maintaining predator functional diversity in farming landscapes in order to suppress different sized pest species for integrated pest control^66,67^.

Camera trap images also indicated that more visits occurred closer to the crop edge, by day and night (Table 2). However, small passerines were not captured on camera traps (Table 3b) and their contribution to prey removal was uncertain. Whilst more prey items were generally removed from feeding stations during the day, a greater number of camera images (mainly of insectivorous bats) were tagged at night. Maximum insectivorous bat activity was recorded in January (which corresponds to young bats becoming volant); yet experimental prey removed from the feeding stations was least in this month. Newly volant bats may not have removed prey from feeding stations because the predation risk was too high, due to reduced crop cover. A similar finding by Nelson and Gillam^68^ showed that during vulnerable life stages (pregnancy and lactation), bats prefer edge habitat due to the reduced predation risk. Furthermore, we hypothesise that, like birds^69,70^, newly volant juvenile bats are at greater risk of predation than adult bats, and that bold risk-taking behaviour in foraging bats increases with age. This may explain the high nocturnal mealworm removal in February and March, but not January.

Images of insectivorous bats did not necessarily reflect prey removal, although the presence of bats in the crop presumably inferred foraging behaviour and it is likely that bats were responsible for some mealworm removal with gleaning bat species present (Kolkert et al. unpublished data). This highlights the fact that artificial prey experiments may not accurately reflect the predation service to be estimated. Bats captured on camera were likely foraging for alternate food resources in the crop i.e. abundant crop insects. This suggested that bats were selecting a preferred food source inside the crop (i.e. moths), consistent with Optimal Foraging Theory (OFT), that predicts when prey is abundant (i.e. ~ 450 species of cotton insects) predators become selective with prey choice. Insectivorous bats in this area are known to consume agricultural arthropods, particularly moths, in cotton crops^24^. Therefore, bats captured on camera were likely hunting moths. This finding suggests the potential for camera traps to infer predation rates of crop pests by insectivorous bats in cropping agroecosystems. Further work is needed to calibrate the number of camera trap images of bats with pest predation and consumption volumes.

Artificial predation experiments are limited as they do not accurately reflect the visual, behavioural or physiological cues relied on to detect prey^71–73^. Although we attempted to account for predator cues by using live mealworms and regionally abundant dead beetles, beetles do not comprise a large component of insectivorous bat diets in crops in this region (H. Kolkert et al., unpublished data) and are less nutritious than mealworms^74^, which may explain why more mealworms were removed, and why the effects of proximity to crop edge were stronger with mealworms. For these reasons we suspect mealworms were the preferred food item for other small insectivorous birds, such as fairywrens, cisticolas and willie wagtails, with larger generalist predators, such as owls, tawny frogmouths, Australian magpies, also consuming beetles. Prey choice and participation in the predation experiment for a variety of predators was likely influenced by the abundant and diverse insect supply in the Bt-cotton^61,72^. It is possible that that attack rates on experimental prey may be under-inflated due to low predation pressure^72^. Nevertheless, we attempted to provide live stimuli and different sized prey to encourage participation in the experiment by a diversity of insectivore predators.

Future studies should aim to test other prey resources (potentially pest species) to further understand foraging mechanisms and prey selection by whole predator communities at the crop edge. Regardless, our camera trap and experimental prey removal results suggest that insectivorous predators forage in crops as predicted by OFT^50^, where the trade-off between resource maximisation and the risk of predation of the predators increased away from the crop edge. Whilst this was also true for bats, it is likely that echolocation constraints also contributed to foraging closer to the crop edge. Knowing that fear of predation, rather than food availability drives the spatial foraging patterns of insectivorous predators close to the crop interface (except for large generalist open space foragers like Australian magpies) can encourage land managers to provide suitable non-crop habitat adjacent to crop edges.

In conclusion, our results showed clear patterns between the removal of sentinel insect prey in crops, proximity to the cotton crop edge and the presence of potential predators. Information should be provided about how to encourage open space generalist predators early in the growing season and cover-dependent small passerines later in the season for pest control of different sized insects. Furthermore, information about the predation gradient from the crop edge and the rate of change in natural pest control with increasing distance into the crop over the growing season can be used to measure the outcome of biodiversity modifications on farms to benefit natural pest control. We conclude that optimal natural pest control in cotton at different phenological stages likely occurs via a diversity of predator functional groups. Maintaining a mix of structural vegetation components adjacent to the crop edge^75^ could buffer the variability in predator communities, particularly small passerines that avoid open habitat.

## Methods

### Ethics

Animal ethics approval was obtained from the University of New England Animal Ethics Committee (approval no. AEC13-060) in addition to a New South Wales (NSW) scientific licence (licence no. SL101296) issued by the NSW Office of Environment and Heritage. All methods were performed in accordance with the relevant guidelines and regulations.

### Study sites

**Figure 3:**
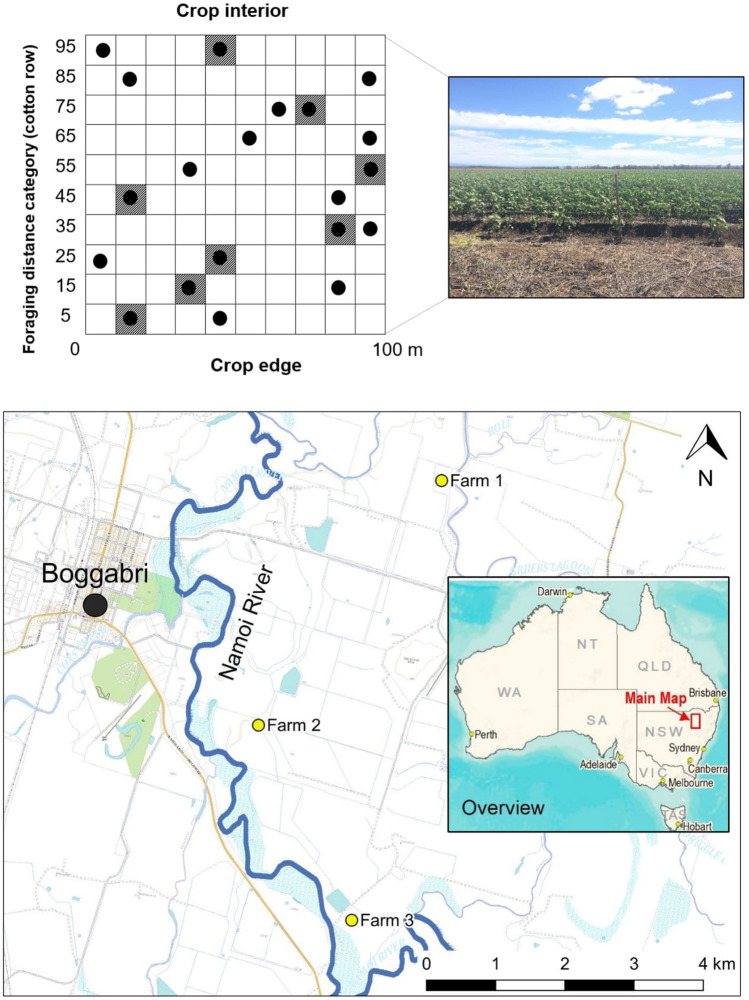
Location and example of the trial design at each farm showing a 100 × 100-m sampling area. The y-axis indicates the foraging distance (cotton row) from the edge of the cotton crop. Circles represent feeding stations (n = 20) and hatched cells indicate the location of a camera trap (n = 8).

This research was conducted during the 2014–15 summer cotton growing season on three irrigated cotton farms near Boggabri (30°43′15.4"S 150°04′52.5"E) in northern NSW, Australia (Fig. 3). The combined farm area totalled 1654 ha, with 925 ha dedicated to irrigated cotton, 185 ha to dryland cotton, 335 ha to grazing and the remainder to houses, outbuildings, roads and irrigation channels. The research took place in three fields of irrigated cotton (one on each farm): 45 ha, 25 ha and 12 ha in size. The distance between each field was 4.0 km, 2.5 km and 6.0 km respectively. Cotton was planted in early October 2014 and no insecticide was applied during the growing season. At these farms (consistent with cotton grown in subtropical zones around Australia), the cotton flowers around December, cotton bolls develop in January and vegetative growth peaks around February. By March the majority of bolls open and crop harvesting occurs between March and April. Irrigated Bollgard II cotton was grown on these farms, planted in a configuration of one row of cotton per metre. Bollgard II cotton (Bt-cotton) contains two genes derived from the soil bacterium, *Bacillus thuringiensis,* producing toxins that are lethal to the larvae of Lepidoptera. About 10% of the cropping area at each farm was dedicated to unsprayed refuge crops (non-Bt, cotton and pigeon pea). Refuge crops delay the resistance of moths to the Bt toxin, by allowing Bt-resistant and Bt-susceptible moths to mate and disperse in crops.

These farms occur in the sub-tropical Brigalow Belt South Bioregion^76^ on black self-mulching clay alluvial plains adjacent to the Namoi River^77^. Remnant vegetation adjacent to each cotton field was predominantly forest, floodplain woodland and semi-arid grassland^78^ most commonly dominated by river red gum (*Eucalyptus camaldulensis*) open woodland associated with the Namoi River and adjacent floodplain. Native vegetation adjacent to each cotton field was unmanaged and extended along the entirety of the Namoi River in the region.

### Trial design

At each farm, a 100 × 100 m (1 ha) sampling area of cotton crop was selected adjacent to remnant vegetation. Each 1 ha sampling area was divided into a 10 × 10 m grid with ten successive predation distances from the crop edge and ten successive bands of cotton, perpendicular to the crop edge. The first predation distance was 5 m from the edge of the crop (fifth row of cotton) with subsequent predation distances at 10-m intervals (15, 25 … 95 m; Fig. 3). At each predation distance, random number generation determined in which band a feeding station was placed. Each feeding station consisted of a plastic green tray (250 × 250 × 40 mm) nailed to a wooden stake, 1 m above the ground in the cotton row and left in situ for the duration of the experiment (total n = 60, 20 sampling points per site with two stations per distance). The possibility of predators learning the location of feeding stations and associating them with a food resource was accounted for by leaving the stations *in-situ* outside of sampling times.

Previously frozen and thawed *Sericesthis geminata* (Scarabaeoidea, Coleoptera, collected in Armidale and Boggabri, NSW) and live mealworms (the larvae of *Tenebrio molitor*; Pisces Enterprises) were placed on each feeding station to simulate a prey resource. Experimental trials indicated that live *Helicoverpa armigera* larvae (Lepidoptera: Noctuidae, a major pest of cotton) could not withstand the heat of the day (≥ 40˚C) on the feeding station. This led to the decision to use live mealworms with a hard exoskeleton and beetles to attract a diversity of predators. *Sericesthis geminata* are common in Boggabri during the cotton growing season and emerge *en masse* and swarm on warm nights. It is plausible that nocturnal fauna including insectivorous bats were attracted to this prey resource.

Each feeding station had 15 mealworms (15–20 mm long, 3 mm diameter) and 10–15 beetles (11–16 mm long). The number of beetles was always consistent within a sampling period, between farms. Prey removal was recorded, and prey replenished every morning and evening within an hour of sunrise and sunset. Prey removal was measured over the cotton growing season between December and March inclusive. Sampling occurred over 29 days (8 consecutive days in January; 7 consecutive days in other months) on three farms day and night. Sampling was missed on eleven occasions due to inclement weather. This resulted in 3260 sampling observations of feeding stations on the three farms across the season.

### Identification of potential predators

Infrared motion-activated trail cameras (ScoutGuard SG550) were deployed at eight feeding stations per sampling area to record the animals visiting feeding stations. When motion was detected, one image per second for 3 secs was captured. Cameras were set to record for the duration of each sampling session and placed in the same position at each subsequent sampling session. Fauna images were identified to species where possible and categorised as day or night.

Bird species richness and abundance undertaken from the crop edge (point counts) over each sampling area for a period of 20 min each morning and evening (within an hour of sunrise and sunset) while replenishing feeding trays (n = 348 sampling events, 29 days by three farms day and night). The order of fields visited for bird observations was randomised. Scats deposited on trays and ant abundance were recorded at each feeding station and plates cleared of scats every morning and afternoon. These methods were used to evaluate the potential role of insectivorous birds and ants as predators of the experimental prey. Climate data from the Bureau of Meteorology (station no. 055202) was recorded each day.

### Data analysis

Data analysis was performed in R. software (version 4.0.2)^79^. We used the glmmTMB package (v1.0.2.1, published 2020–07-01) to fit binomially distributed data using maximum likelihood estimation^80^. To determine the probability of a predation event, the number of prey removed (mealworms or beetles) of the pre-set number on offer was the response variable. Generalised linear mixed-effects models (GLMMs) were fitted with a beta-binomial distribution that captured overdispersion in the binomially distributed data and accounted for varying numbers of prey at feeding stations.

We tested whether the probability of a predation event was affected by the following fixed effects and interactions: time of day (day or night), month (December to March) and foraging distance (i.e. proximity to cotton edge; 5, 15, 25 … 95 m). Farm (three levels) and feeding stations were treated as random effects. A separate conditional model was created for each of the two prey resources (mealworms and beetles). A zero-inflation parameter was applied to the beetle model, where the probability of producing a structural zero was a function for all observations (ziformula ≃ 1). The zero-inflation parameter described the probability of observing an extra (i.e. structural) zero that was not generated by the conditional model. Predicted marginal effects (predicted probabilities for logistic models) of the mealworm removal rate from the crop edge were made using the ‘ggeffects’ package (v0.7.0, published 2018–11-17)^81^, taking random effects into account. Wald-type 95% confidence intervals for predicted average count were calculated. Residuals and diagnostics of models were checked using the DHARMa package (v0.2.0, published 2018–06-06), a simulation-based approach for checking model assumptions and diagnostics^82^. We identified the best fitting model based on Dharma diagnostics. The EMMEANS package (v1.5.1) was used to calculate estimated marginal means using the Tukey method to determine which pairwise differences were significant^83^.

To derive an estimate of the change in predation risk, we adopted an odds-based approach where odds-ratios (*OR*) were calculated by exponentiating the model coefficients (β). Odds-ratios were converted to a percent and calculated as (1 – exp(β) × 100) where *OR* ≤ 1, and (exp(β) – 1 × 100) where *OR* > 1. The resulting *OR* then describes the factor by which predation odds change for a 1-unit increase in the corresponding factor^84^.

Camera images with an animal present were tagged as a visitation event. Visitation events were quantified in two ways: (1) counts of visitations at each feeding station (or in the camera field of view for bats) were calculated as camera trap images per hour (CT/h), and (2) the length of time of visitations at each feeding station was calculated as an activity index (AI/h). Each method was adjusted for the sampling time at each location and standardised by the number of tagged images (1) or hours of recording (2). Visitation events were then allocated to a distance from the crop edge (close: 5–45 m; far: 55–95 m).

Bird abundance, bird richness, ant abundance and scat abundance data for every sampling observation and site were tabulated. Pearson correlation coefficients (r) were calculated between climate data (minimum and maximum temperature, daily rainfall, daily evaporation, maximum daily wind speed) and the predation rate of mealworms, beetles and total predation rate (combined mealworm and beetle predation).

## Supplementary Information

Supplementary Information.

## Data Availability

Should the manuscript be accepted, the data supporting the results will be archived in an appropriate public repository such as Dryad or Figshare and the data DOI will be included at the end of the article.
