# Supplementary material for: Prey removal in cotton crops next to woodland reveals periodic diurnal and nocturnal invertebrate predation gradients from the crop edge by birds and bats
Source: Sci Rep. 2021 Mar 4;11:5256. doi: 10.1038/s41598-021-84633-8 (PMC7970855; doi:10.1038/s41598-021-84633-8)
Supplement: Supplementary file 1 — Supplementary Information. [file 41598_2021_84633_MOESM1_ESM.docx]

**Title:** Prey removal in cotton crops next to woodland reveals periodic diurnal and nocturnal invertebrate predation gradients from the crop edge by birds and bats

**Authors:** Heidi L. Kolkert^1^*, Rhiannon Smith^1^, Romina Rader^1^ and Nick Reid^1^

^1^Ecosystem Management, School of Environmental and Rural Science, University of New England, NSW 2351, Australia

**Correspondence**:

* Ecosystem Management, School of Environmental and Rural Science, University of New England, NSW 2351, Australia

hkolker2@une.edu.au

ph: +61 418324136

Heidi Kolkert ORC ID: https://orcid.org/0000-0003-0817-143X

Rhiannon Smith ORC ID: <https://orcid.org/0000-0002-6375-5684>

Romina Rader ORC ID: https://orcid.org/0000-0001-9056-9118

Nick Reid ORC ID: https://orcid.org/0000-0002-4377-9734

# Supplementary material

**Table S1. Results of the generalized linear mixed models (glmmTMB) using a beta-binomial distribution on factors affecting the probability of a predation event of mealworm and beetle prey. Standard error (se), lower confidence interval (l.ci) and upper confidence interval (u.ci). The zero inflated model predicts non-occurrence of the outcome.**

| **Conditional model: mealworms** | | | | |  |  |  |
| --- | --- | --- | --- | --- | --- | --- | --- |
|  | Estimate | se | z value | p-value | |  |  |
|  |  |  |  |  | |  |  |
| (Intercept) | -1.825 | 0.288 | -6.338 | **0.000** | |  |  |
| Time NIGHT | -1.692 | 0.471 | -3.595 | **0.000** | |  |  |
| Cotton row | -0.001 | 0.004 | -0.138 | 0.890 | |  |  |
| Month Feb | 0.707 | 0.321 | 2.201 | **0.028** | |  |  |
| Month Jan | 0.152 | 0.345 | 0.439 | 0.660 | |  |  |
| Month March | 0.688 | 0.315 | 2.183 | **0.029** | |  |  |
| Time NIGHT: Cotton row | 0.005 | 0.008 | 0.596 | 0.551 | |  |  |
| Time NIGHT: Month Feb | 1.768 | 0.565 | 3.128 | **0.002** | |  |  |
| Time NIGHT: Month Jan | 0.592 | 0.636 | 0.931 | 0.352 | |  |  |
| Time NIGHT: Month March | 0.303 | 0.582 | 0.522 | 0.602 | |  |  |
| Cotton row: Month Feb | -0.002 | 0.006 | -0.284 | 0.776 | |  |  |
| Cotton row: Month Jan | -0.020 | 0.007 | -2.978 | **0.003** | |  |  |
| Cotton row: Month March | -0.008 | 0.006 | -1.486 | 0.137 | |  |  |
| Time NIGHT: Cotton row :Month Feb | -0.026 | 0.010 | -2.593 | **0.010** | |  |  |
| Time NIGHT: Cotton row: Month Jan | 0.005 | 0.012 | 0.381 | 0.703 | |  |  |
| Time NIGHT: Cotton row: Month March | 0.012 | 0.010 | 1.185 | 0.236 | |  |  |
|  |  |  |  |  | |  |  |
|  |  |  |  |  | |  |  |
| **Conditional model: beetles** | | | | |  |  |  |
| (Intercept) | 0.236 | 0.348 | 0.677 | 0.498 | |  |  |
| Time NIGHT | -2.403 | 0.490 | -4.904 | **0.000** | |  |  |
| Cotton row | 0.008 | 0.006 | 1.321 | 0.186 | |  |  |
| Month Feb | -1.740 | 0.431 | -4.039 | **0.000** | |  |  |
| Month Jan | -3.216 | 0.516 | -6.237 | **0.000** | |  |  |
| Month March | -2.665 | 0.472 | -5.640 | **0.000** | |  |  |
| Time NIGHT: Cotton row | -0.004 | 0.009 | -0.450 | 0.653 | |  |  |
| Time NIGHT: Month Feb | 1.449 | 0.666 | 2.176 | **0.030** | |  |  |
| Time NIGHT: Month Jan | 2.294 | 0.795 | 2.886 | **0.004** | |  |  |
| Time NIGHT: Month March | 2.442 | 0.671 | 3.637 | **0.000** | |  |  |
| Cotton row: Month Feb | -0.011 | 0.008 | -1.403 | 0.161 | |  |  |
| Cotton row: Month Jan | -0.003 | 0.009 | -0.376 | 0.707 | |  |  |
| Cotton row: Month March | -0.003 | 0.008 | -0.343 | 0.732 | |  |  |
| Time NIGHT: Cotton row: Month Feb | 0.003 | 0.012 | 0.283 | 0.777 | |  |  |
| Time NIGHT: Cotton row: Month Jan | -0.013 | 0.015 | -0.913 | 0.361 | |  |  |
| Time NIGHT: Cotton row: Month March | 0.005 | 0.011 | 0.412 | 0.680 | |  |  |
| **Zero-inflation model** |  |  |  |  | |  |  |
| (Intercept) | -0.127 | 0.151 | 0.848 | 0.397 | |  |  |

**Table S2: Odds ratios estimate of mealworm and beetle predation with Wald’s 2.5% and 97.5% confidence limits. Odds ratios for the conditional model were calculated by exponentiating the coefficients.**

| Mealworms | | | | |
| --- | --- | --- | --- | --- |
|  | **Odds Ratio** | **2.5%** | **97.5%** | **Estimate** |
| cond. (Intercept) |  |  |  |  |
| cond. Time NIGHT | 0.161 | 0.092 | 0.284 | -1.825 |
| cond. Cotton row | 0.184 | 0.073 | 0.463 | -1.692 |
| cond. Month Feb | 0.999 | 0.991 | 1.010 | -0.001 |
| cond. Month Jan | 2.030 | 1.080 | 3.810 | 0.707 |
| cond. Month March | 1.160 | 0.592 | 2.290 | 0.152 |
| cond. Time NIGHT: Cotton row | 1.990 | 1.070 | 3.690 | 0.688 |
| cond. Time NIGHT: Month Feb | 1.000 | 0.989 | 1.020 | 0.005 |
| cond. Time NIGHT: Month Jan | 5.860 | 1.940 | 17.700 | 1.768 |
| cond. Time NIGHT: Month March | 1.810 | 0.520 | 6.290 | 0.592 |
| cond. Cotton row: Month Feb | 1.350 | 0.433 | 4.240 | 0.303 |
| cond. Cotton row: Month Jan | 0.998 | 0.988 | 1.010 | -0.002 |
| cond. Cotton row: Month March | 0.980 | 0.967 | 0.993 | -0.020 |
| cond. Time NIGHT: Cotton row: Month Feb | 0.992 | 0.981 | 1.000 | -0.008 |
| cond. Time NIGHT: Cotton row: Month Jan | 0.975 | 0.956 | 0.994 | -0.026 |
| cond. Time NIGHT: Cotton row: Month March | 1.000 | 0.982 | 1.030 | 0.005 |
| cond. Std.Dev. Farm.(Intercept) | 1.010 | 0.992 | 1.030 | 0.012 |
| Beetles | | | | |
| cond. (Intercept) | 0.386 | 0.640 | 2.506 | 1.266 |
| cond. Time NIGHT | 0.146 | 0.035 | 0.236 | 0.090 |
| cond. Cotton row | 1.003 | 0.996 | 1.019 | 1.008 |
| cond. Month Feb | 0.330 | 0.075 | 0.408 | 0.176 |
| cond. Month Jan | 0.078 | 0.015 | 0.110 | 0.040 |
| cond. Month March | 0.132 | 0.028 | 0.176 | 0.070 |
| cond. Time NIGHT: Cotton row | 1.000 | 0.980 | 1.013 | 0.996 |
| cond. Time NIGHT: Month Feb | 2.578 | 1.155 | 15.700 | 4.258 |
| cond. Time NIGHT: Month Jan | 6.325 | 2.088 | 47.098 | 9.918 |
| cond. Time NIGHT: Month March | 7.089 | 3.083 | 42.836 | 11.491 |
| cond. Cotton row: Month Feb | 0.994 | 0.975 | 1.004 | 0.989 |
| cond. Cotton row: Month Jan | 1.001 | 0.980 | 1.014 | 0.997 |
| cond. Cotton row: Month March | 1.001 | 0.981 | 1.013 | 0.997 |
| cond. Time NIGHT: Cotton row: Month Feb | 1.000 | 0.981 | 1.026 | 1.003 |
| cond. Time NIGHT: Cotton row: Month Jan | 0.983 | 0.959 | 1.015 | 0.987 |
| cond. Time NIGHT: Cotton row: Month March | 1.001 | 0.982 | 1.028 | 1.005 |
| cond. Std.Dev. Farm.(Intercept) | 0.386 | 1.000 | Inf | 1.000 |
| zi.(Intercept) | 0.146 | 1.000 | Inf | 1.002 |
|  |  |  |  |  |

**Table S3: Estimated predation of mealworms at incremental 5m distances from the crop edge to 95m inside the crop. Predicted estimates take into account random effects, showing; lower confidence interval (l.ci), upper confidence interval (u.ci). Standard errors (se) are on link-scale (untransformed).**

| **Population average over day and night** | | | | | | | | | |  |
| --- | --- | --- | --- | --- | --- | --- | --- | --- | --- | --- |
| **Day** |  |  |  |  | **Night** |  |  |  |  |  |
| Cotton row | predicted | se | l.ci | u.ci | Cotton row | predicted | se | l.ci | u.ci |  |
| 5 | 0.15 | 0.35 | 0.080 | 0.26 | 5 | 0.06 | 0.42 | 0.030 | 0.13 |  |
| 15 | 0.13 | 0.33 | 0.070 | 0.22 | 15 | 0.05 | 0.38 | 0.030 | 0.11 |  |
| 35 | 0.09 | 0.31 | 0.050 | 0.15 | 35 | 0.04 | 0.34 | 0.020 | 0.08 |  |
| 55 | 0.06 | 0.32 | 0.030 | 0.11 | 55 | 0.03 | 0.35 | 0.020 | 0.07 |  |
| 65 | 0.05 | 0.34 | 0.030 | 0.09 | 65 | 0.03 | 0.38 | 0.010 | 0.06 |  |
| 95 | 0.03 | 0.44 | 0.010 | 0.06 | 95 | 0.02 | 0.51 | 0.010 | 0.06 |  |
|  |  |  |  |  |  |  |  |  |  |  |
|  | | | | | | | | | |  |
| **Day** |  |  |  |  | **Night** | |  |  |  |  |
| Cotton row | predicted | se | l.ci | u.ci | Cotton row | | predicted | se | l.ci | u.ci |
| **December** |  |  |  |  | **December** | |  |  |  |  |
| 5 | 0.150 | 0.350 | 0.080 | 0.250 | 5 | | 0.030 | 0.470 | 0.010 | 0.080 |
| 25 | 0.140 | 0.320 | 0.080 | 0.240 | 25 | | 0.030 | 0.390 | 0.020 | 0.070 |
| 55 | 0.140 | 0.300 | 0.080 | 0.230 | 55 | | 0.040 | 0.340 | 0.020 | 0.070 |
| 95 | 0.140 | 0.350 | 0.070 | 0.240 | 95 | | 0.040 | 0.440 | 0.020 | 0.100 |
| **January** |  |  |  |  | **January** | |  |  |  |  |
| 5 | 0.150 | 0.350 | 0.080 | 0.260 | 5 | | 0.060 | 0.420 | 0.030 | 0.130 |
| 25 | 0.110 | 0.320 | 0.060 | 0.180 | 25 | | 0.050 | 0.360 | 0.020 | 0.090 |
| 55 | 0.060 | 0.320 | 0.030 | 0.110 | 55 | | 0.030 | 0.350 | 0.020 | 0.070 |
| 95 | 0.030 | 0.440 | 0.010 | 0.060 | 95 | | 0.020 | 0.510 | 0.010 | 0.060 |
| **February** |  |  |  |  | **February** | |  |  |  |  |
| 5 | 0.240 | 0.340 | 0.140 | 0.380 | 5 | | 0.240 | 0.340 | 0.140 | 0.380 |
| 25 | 0.240 | 0.310 | 0.150 | 0.360 | 25 | | 0.170 | 0.310 | 0.100 | 0.270 |
| 55 | 0.230 | 0.290 | 0.140 | 0.350 | 55 | | 0.090 | 0.310 | 0.050 | 0.160 |
| 95 | 0.220 | 0.330 | 0.130 | 0.350 | 95 | | 0.040 | 0.400 | 0.020 | 0.080 |
| **March** |  |  |  |  | **March** | |  |  |  |  |
| 5 | 0.250 | 0.330 | 0.140 | 0.380 | 5 | | 0.080 | 0.370 | 0.040 | 0.150 |
| 25 | 0.210 | 0.310 | 0.130 | 0.330 | 25 | | 0.090 | 0.330 | 0.050 | 0.160 |
| 55 | 0.170 | 0.300 | 0.100 | 0.270 | 55 | | 0.110 | 0.300 | 0.070 | 0.190 |
| 95 | 0.120 | 0.350 | 0.070 | 0.220 | 95 | | 0.140 | 0.350 | 0.080 | 0.250 |

**Table S4:** Pearson’s correlation and p-value between prey items removed (total prey, beetle and mealworms), climate variables, bird abundance (bird abun), bird richness (bird rich), ant abundance (ant) and scat abundance (scat).

| **Pearson's r** | Total prey | Beetles | Mealworms | Temp (min) ˚C | Temp (max) ˚C | Rain (mm) | Evap (mm) | Wind gusts max (km/hr) | Bird rich | Bird abun | Ant |
| --- | --- | --- | --- | --- | --- | --- | --- | --- | --- | --- | --- |
| Total prey |  |  |  |  |  |  |  |  |  |  |  |
| Beetles | 0.69 |  |  |  |  |  |  |  |  |  |  |
| Mealworms removed | 0.83 | 0.23 |  |  |  |  |  |  |  |  |  |
| Temp (min) ˚C | 0.13 | 0.04 | 0.12 |  |  |  |  |  |  |  |  |
| Temp (max) ˚C | 0.05 | 0.02 | -0.02 | 0.05 |  |  |  |  |  |  |  |
| Rain (mm) | -0.04 | -0.07 | 0 | 0.38 | 0.13 |  |  |  |  |  |  |
| Evaporation (mm) | 0.11 | 0.13 | -0.03 | -0.19 | 0.31 | -0.46 |  |  |  |  |  |
| Wind gusts max (km/hr) | 0.14 | 0.18 | -0.04 | 0.18 | 0.47 | -0.15 | 0.28 |  |  |  |  |
| Bird richness | 0.00 | -0.04 | 0.05 | -0.04 | -0.04 | -0.07 | 0.01 | -0.08 |  |  |  |
| Bird abundance | 0.03 | -0.03 | 0.07 | -0.04 | -0.05 | -0.08 | -0.01 | -0.05 | 0.85 |  |  |
| Ants | 0.05 | -0.07 | 0.16 | -0.14 | -0.19 | -0.09 | -0.14 | -0.23 | -0.07 | -0.11 |  |
| Scats | 0.15 | 0.09 | 0.16 | 0.13 | 0.03 | -0.08 | 0.07 | -0.05 | 0.1 | 0.06 | 0.06 |
|  |  |  |  |  |  |  |  |  |  |  |  |
| **p-values** |  |  |  |  |  |  |  |  |  |  |  |
| Total prey |  |  |  |  |  |  |  |  |  |  |  |
| Beetles | 0.00 |  |  |  |  |  |  |  |  |  |  |
| Mealworms removed | 0.00 | 0.00 |  |  |  |  |  |  |  |  |  |
| Temp (min) ˚C | 0.00 | 0.02 | 0.00 |  |  |  |  |  |  |  |  |
| Temp (max) ˚C | 0.00 | 0.18 | 0.30 | 0.01 |  |  |  |  |  |  |  |
| Rain (mm) | 0.02 | 0.00 | 0.90 | 0.00 | 0.00 |  |  |  |  |  |  |
| Evaporation (mm) | 0.00 | 0.00 | 0.09 | 0.00 | 0.00 | 0.00 |  |  |  |  |  |
| Wind gusts max (km/hr) | 0.00 | 0.00 | 0.01 | 0.00 | 0.00 | 0.00 | 0.00 |  |  |  |  |
| Bird richness | 0.95 | 0.02 | 0.00 | 0.04 | 0.01 | 0.00 | 0.44 | 0.00 |  |  |  |
| Bird abundance | 0.15 | 0.09 | 0.00 | 0.01 | 0.01 | 0.00 | 0.54 | 0.01 | 0.00 |  |  |
| Ants | 0.00 | 0.00 | 0.00 | 0.00 | 0.00 | 0.00 | 0.00 | 0.00 | 0.00 | 0.00 |  |
| Scats | 0.00 | 0.00 | 0.00 | 0.00 | 0.08 | 0.00 | 0.00 | 0.00 | 0.00 | 0.00 | 0.00 |


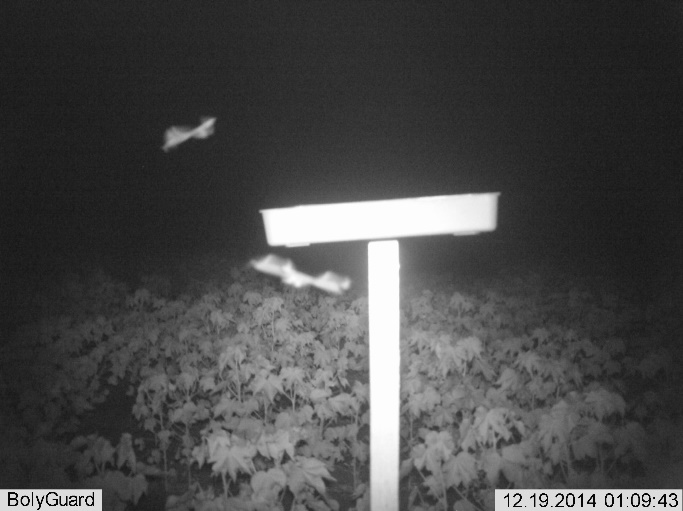


**Fig S1: Camera trap images of predators visiting the feeding stations during the trial. Top-left clockwise: insectivorous bats mid-flight, *Ninox boobook* (southern boobook), *Rhipidura leucophrys* (willie wagtail) and *Gymnorhina tibicen* (Australian magpie).**
